# The Role of Isothiocyanates as Cancer Chemo-Preventive, Chemo-Therapeutic and Anti-Melanoma Agents

**DOI:** 10.3390/antiox8040106

**Published:** 2019-04-18

**Authors:** Melina Mitsiogianni, Georgios Koutsidis, Nikos Mavroudis, Dimitrios T. Trafalis, Sotiris Botaitis, Rodrigo Franco, Vasilis Zoumpourlis, Tom Amery, Alex Galanis, Aglaia Pappa, Mihalis I. Panayiotidis

**Affiliations:** 1 Department of Applied Sciences, Northumbria University, Newcastle Upon Tyne NE1 8ST, UK; melina.mitsiogianni@northumbria.ac.uk (M.M.); georgios.koutsidis@northumbria.ac.uk (G.K.); 2 Department of Food and Nutritional Sciences, University of Reading, Reading RG6 6AP, UK; nikos.mavroudis@reading.ac.uk; 3 Laboratory of Pharmacology, Unit of Clinical Pharmacology, Medical School, National and Kapodistrian University of Athens, 11527 Athens, Greece; dtrafal@med.uoa.gr; 4 Second Department of Surgery, Democritus University of Thrace, 68100 Alexandroupolis, Greece; smpotait@med.duth.gr; 5 Redox Biology Centre, University of Nebraska-Lincoln, Lincoln, NE 68588, USA; rodrigo.franco@unl.edu; 6 School of Veterinary Medicine and Biomedical Sciences, University of Nebraska-Lincoln, Lincoln, NE 68583, USA; 7 Institute of Biology, Medicinal Chemistry and Biotechnology, National Hellenic Research Foundation, 11635 Athens, Greece; vzub@eie.gr; 8 The Watrercress Company/The Wasabi Company, Waddock, Dorchester, Dorset DT2 8QY, UK; tom.amery@thewatercresscompany.com; 9 Department of Molecular Biology and Genetics, Democritus University of Thrace, 68100 Alexandroupolis, Greece; agalanis@mbg.duth.gr (A.G.); apappa@mbg.duth.gr (A.P.)

**Keywords:** isothiocyanates, glucosinolates, chemo-therapy, cruciferous vegetables, skin cancer, malignant melanoma, cell cycle, growth arrest

## Abstract

Many studies have shown evidence in support of the beneficial effects of phytochemicals in preventing chronic diseases, including cancer. Among such phytochemicals, sulphur-containing compounds (e.g., isothiocyanates (ITCs)) have raised scientific interest by exerting unique chemo-preventive properties against cancer pathogenesis. ITCs are the major biologically active compounds capable of mediating the anticancer effect of cruciferous vegetables. Recently, many studies have shown that a higher intake of cruciferous vegetables is associated with reduced risk of developing various forms of cancers primarily due to a plurality of effects, including (i) metabolic activation and detoxification, (ii) inflammation, (iii) angiogenesis, (iv) metastasis and (v) regulation of the epigenetic machinery. In the context of human malignant melanoma, a number of studies suggest that ITCs can cause cell cycle growth arrest and also induce apoptosis in human malignant melanoma cells. On such basis, ITCs could serve as promising chemo-therapeutic agents that could be used in the clinical setting to potentiate the efficacy of existing therapies.

## 1. Introduction

Nutrition is known to play an important role in human health, primarily in the context of preventing chronic disease development including cancer. As such, it can have a positive impact in health outcomes, thus improving quality of life [1,2]. Overall, the protective role from consuming plant-derived foods has been documented and well established in numerous studies. In particular, the potential of fruits and vegetables in disease prevention was documented in the case of phytochemicals, the bioactive components of plant-derived foods capable of exhibiting a wide range of biological activities including anti-inflammatory, anti-infectious, anti-oxidant and anti-cancer [3,4,5,6,7]. Furthermore, evidence also shows that these compounds can modulate gene expression through epigenetic mechanisms involving DNA methylation, histone modification and modulation of miRNAs [8,9]. However, their prevailing anti-oxidant capacity is of particular importance given that oxidative stress (and, consequently, accumulation of free radicals) plays a major role in ageing and other chronic diseases, including heart disease, cognitive disorders and cancer. Briefly, an imbalance in the equilibrium of antioxidants and free radicals (of oxygen and/or nitrogen species) can result in DNA damage and genomic instability, thus increasing the risk for disease development and mortality [10,11,12,13]. To these ends, phytochemicals have been shown to contribute to the detoxification of the body and thus have a positive impact on human health and disease prevention [13,14,15]. In the context of cancer chemo-therapy, the use of agents (synthetic, natural or biological) is utilized in order to reverse, suppress or prevent the multi-stage process of carcinogenesis [16,17]. On the other hand, natural products possess pleiotropic mechanisms responsible for their health benefits, including prevention of cancer development [7,14,15]. Phytochemicals are generally categorized according to their chemical properties and functional groups into carotenoids, phenolics, nitrogen-containing (e.g., alkaloids, amines) and organosulfur compounds (e.g., isothiocyanates and allyl sulphides such as allicin) [18]. Especially, sulfur-containing substances (isothiocyanates (ITCs)) have raised scientific interest with their unique properties in cancer prevention and treatment.

Glucosinolates (GLs) are an important group of phytochemicals abundant in vegetables of the Brassicaceae family, including broccoli, watercress, brussels sprouts, cabbage, horseradish, cauliflower, mustard, etc. [19]. They all share a common core structure consisting of a β-thioglucosilated moiety linked to a sulfonated aldoxime and a variable aglucone side chain (R), derived from eight selected amino acids (alanine, leucine, isoleucine, valine, phenylalanine, tyrosine, tryptophan and methionine and possibly glutamic acid) [20,21]. According to the amino acid precursors used at the early stages of their biosynthesis, they are mainly categorized as aliphatic, aromatic, heterocyclic (indoles), benzoate, multiple glycosylated and sulfur-containing GLs [22,23].

Their content and type in plants vary between and within the family members [23]. It is mainly dependant on environmental factors like cultivation, climate, region, genotype, the part of the plant and its developmental stage [24,25,26,27]. To date, about 130 different GL structures have been identified [28]. However, a more detailed review of the literature led to the characterization of a greater number of structures, thus it is currently considered that the number reaches 200 individual structures [23]. Upon hydrolysis, they give rise to various bioactive compounds, including ITCs, responsible for the health benefits provided through the consumption of these cruciferous vegetables [29,30]. ITCs are compounds characterised by an R–N=C=S structure, where R is a variable side chain consisting of alkyl or allyl groups. They are highly reactive electrophiles that interact reversibly with the thiol group of cystine residues to form dithiocarbamates [31].

In general, ITCs are mainly responsible for the flavour and odour of cruciferous vegetables and exert a wide range of bioactivities from defence against pathogens (in *Brassica* vegetables) for the prevention of cancer development [32]. Among them, their anti-microbial, anti-oxidant and anti-inflammatory properties are of particular importance. More specifically, the anti-microbial activity of ITCs has been documented in a number of studies, suggesting possible use of these compounds as natural antibiotic agents, food additives and/or pesticides [33,34,35,36,37,38,39]. It has been reported that they have both bacteriostatic and bactericidal potencies and many mechanisms have been suggested to mediate these properties. Briefly, ITCs exert anti-microbial activities through disruption of the cell membrane, deregulation of enzymatic processes and induction of heat-shock proteins as well as oxidative stress [33]. In addition, ITCs can also act as indirect antioxidants by activating the Nrf2-dependent pathway [40,41]. To this end, a study by McWalter et al., 2004 has shown that ITCs increased the expression of detoxifying enzymes in both wild-type mice and mouse cell lines, but not in Nrf2-knockdown ones [42]. Moreover, a microarray-based expression profile analysis revealed Nrf2-mediated elevation of antioxidant proteins and metabolizing enzymes in hepatocellular carcinoma (HepG2) cells treated with wasabi-derived sulforaphane (SFN) and SFN analogues, highlighting the importance of this pathway in inducing an antioxidant response [43]. However, a constitutive activation of this pathway could be potentially advantageous for cancer cells by creating an environment that favours cell survival and growth. In addition, the persistent Nrf2 activity can interfere with the metabolic process of some anti-cancer drugs, leading to chemo-resistance [44,45,46]. Overall, it seems that although ectopic expression of Nrf2 could be detrimental in fully-developed cancers, its transient activation in healthy individuals can exert a protective effect [47].

**Figure 1:**
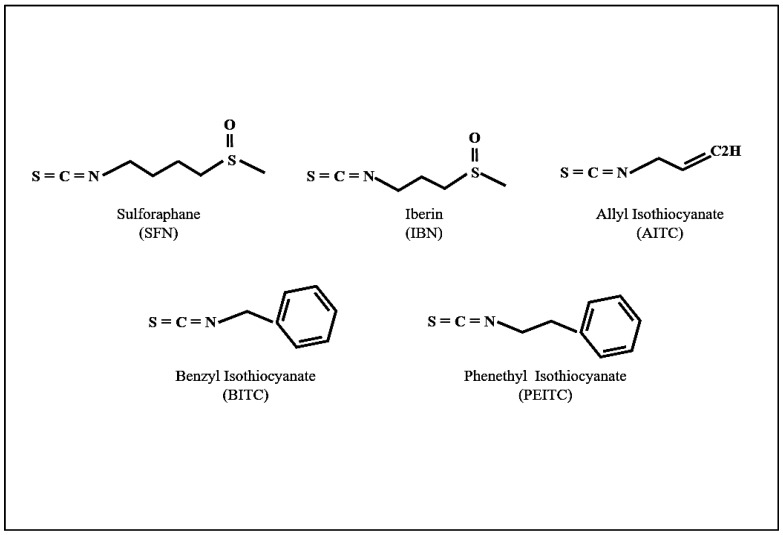
The structures of major isothiocyanates (ITCs).

Apart from its role in anti-oxidant processes, Nrf2 can also mediate an anti-inflammatory response through the transcriptional factor nuclear factor-kappa B (NF-κB) signalling pathway, although the exact mechanism has not yet been elucidated [48,49,50]. On another note, ITCs can also repress the inflammatory process by inhibiting NF-κB which, in turn, regulates the expression of pro-inflammatory and anti-apoptotic proteins [51,52,53]. Moreover, other mechanisms (independent of Nrf2/NF-κB) have also been shown to mediate ITC-induced anti-inflammatory properties including epigenetic alterations [54,55]. For example, it has been shown that SFN suppresses histone deacetylase (HDAC) activity while increasing DNA methyltransferase 1 (DNMT1) expression, thus blocking lipopolysaccharide (LPS)-induced pro-inflammatory cytokine formation in porcine monocyte-derived dendritic cells [55]. Another novel anti-inflammatory role of SFN has been proposed recently, showing that it inhibits the formation of multiple inflammasomes and thus showing an action against inflammasome-originated diseases [56]. Finally, another mechanism involves the binding to the nucleophilic N-terminal proline residue of the macrophage migration inhibitory factor (MIF), thus modifying its structure and preventing its interaction with extracellular receptors and other protein targets. MIF is a pro-inflammatory cytokine with pro-tumourigenic, pro-angiogenic and anti-apoptotic properties. As such, it is involved in various inflammatory diseases, like rheumatoid arthritis and atherosclerosis, in addition to being implicated at different stages of tumour development, including proliferation and angiogenesis [57,58,59]. Consequently, a number of studies have supported the role of ITCs, especially sulforaphane (SFN), iberin (IBN), allyl-ITC (AITC), benzyl-ITC (BITC) and phenethyl-ITC (PEITC) (Figure 1), in cancer prevention and chemotherapy [60,61,62,63,64], topics which will be discussed in more detail below.

### 1.1. GLs-Myrosinase System

**Figure 2:**
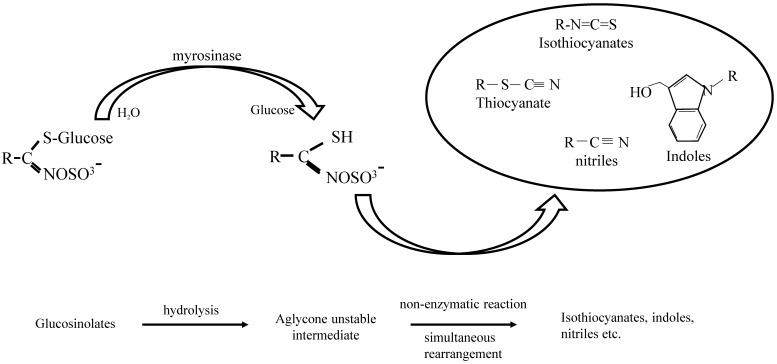
Hydrolysis of glucosinolates by myrosinase.

GL hydrolysis is catalysed by an enzyme called myrosinase, which was discovered in 1839 by Bussy as a protein necessary for the release of essential oil from mustard seed [65]. Disruption of the plant by chewing or cutting leads to the release of myrosinase, a β-thioglucosidase, and brings it into contact with their substrates where it breaks down the sulfur group of the glucosidic bond [21,66]. It is mainly considered to be located in idioblastic cells (as opposed to GLs which are located in different cells inside the plant), although more studies are needed to elucidate their compartmentalization within plants [67]. For instance, in the model species *Arapidopsis thaliana*, GLs were found in S-cells (located between the phloem and the endoderm), which are in close proximity to myrosin cells (located at the phloem parenchyma) [67,68,69]. This compartmentalization acts as a barrier that allows GLs to degrade only when the plant is under stress conditions, such as during a pathogen attack or tissue disruption. Activation of the myrosinase–glucosinolate system (also known as mustard oil bomb) results in the formation of diverse chemically and biologically distinct compounds including thiocyanates, isothiocyanates, nitriles and indoles [32,70,71] (Figure 2). Usually, a ‘Lossen’-like rearrangement occurs that promotes formation of ITCs [21,71,72,73]. At the end of this process, the outcome is largely dependent on various other factors like pH, temperature and availability of ferrous ions, epithiospecifier proteins (ESP), presence of proteins that interact with myrosinase as well as type of GLs [70,74,75]. Other factors that affect the content of GLs (and, consequently, their concentration and intake) include food processing, cultivation, storage and post-harvest processing [76,77,78,79]. Moreover, lower ITC levels can be produced by other mechanisms, including hydrolysis of GLs by bacterial myrosinases. For example, in humans, after consumption of cooked vegetables the formation of ITCs is promoted by the microbiome in the intestinal tract after destruction of endogenous myrosinase due to food preparation; however, this process is poor, resulting in a much lower yield of ITCs [71,80]. Once ITCs are formed in the gut, they enter the cell passively where they bind with high affinity and reversibly to glutathione (GSH) leading to their metabolism (and consequently elimination) through the mercapturic acid pathway. Briefly, ITCs are diffused into the blood circulation through the gastrointestinal tract where they conjugate with GSH, a reversible reaction catalyzed by glutathione S-transferases (GSTs). Enzymatic modifications of the GSH moiety take place in the liver with the final N-acetylcysteine-(NAC-)conjugate being produced in the kidneys, where it is actively secreted into the urine [31,66,70,81].

### 1.2. Anti-Cancer Properties

**Figure 3:**
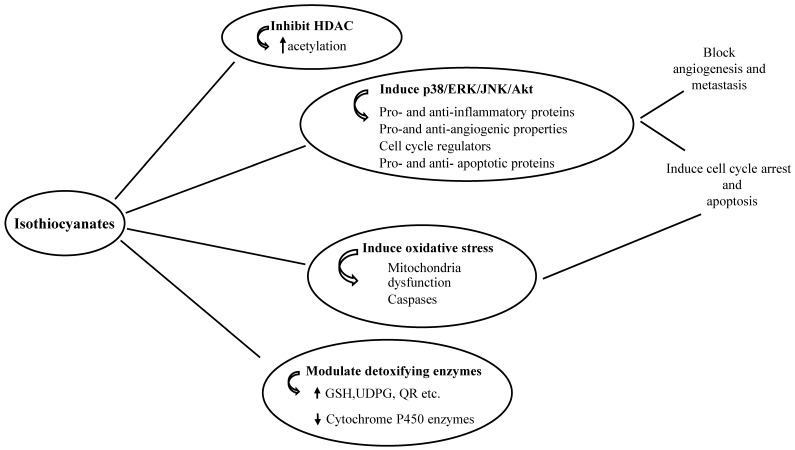
Schematic representation of the proposed molecular pathways targeted by isothiocyanates (ITCs), glutathione (GSH), uridine 5′-diphospho-glucuronosyltransferase (UDPG), quinone reductase (QR).

ITCs exert their anti-tumour activities through various mechanisms, including (i) modulation of phase I and II enzymes, (ii) inhibition of cell growth by causing cell cycle arrest and inducing cell death, (iii) prevention of metastasis and angiogenesis and (iv) regulation of the epigenetic machinery (Figure 3).

#### 1.2.1. Inhibition of Phase I and Induction of Phase II Enzymes

Phase I and II enzymes are important mediators of the detoxification process in the human body. They interact with various chemicals, such as toxins and carcinogens, playing an important role in their metabolism and excretion from the body. Cytochrome P450 (CYP450) enzymes are the most important phase I enzymes, which either deactivate or, in some cases, activate pro-carcinogens to their active form. On the other hand, phase II enzymes exert a predominantly protective role as their interaction with carcinogens increase their elimination from the body. The most important phase II enzymes are glutathione S-transferases (GSTs), uridine 5′-diphospho-glucuronosyltransferase (UDP)-glucuronosyl transferase, nicotinamide adenine dinucleotide phosphate (NADPH), quinine reductases and glutamate cysteine ligase. In general, it is proposed that ITCs downregulate phase I enzymes to inhibit carcinogen activation, while upregulating phase II enzymes to enhance detoxification and prevent reactive oxygen species (ROS)-induced damage [82,83]. SFN (3 mg/kg and 12 mg/kg) has been shown to regulate the activity of CYP2B, CYP3A2 and CYP1A2 enzymes and induce the expression of quinone and glutathione reductase in rats [84]. In line, PEITC (150 μmol/kg) increases the expression of UDP-glucuronosyltransferase UGT1A6 (uridine diphosphate glucuronosyltransferase 1 family, polypeptide A6) and also modulates CYP2B15 activity while decreases nicotinamide N-methyltransferase (NNMT) levels in rats [85]. Both BITC and PEITC were found to prevent nicotine (BITC and PEITC at 25 μM and 3 μM respectively) and tobacco-specific nitrosamine 4-(methylnitrosamino)-1-(3-pyridyl)-1-butanone (NNK) (BITC and PEITC at 1 μM and 0.3 μM, respectively) metabolism by inhibiting the activation of CYP2A6 and CYP2A13, respectively, in an NADPH-dependent manner [86]. Moreover, SFN (10 μM) increased the activity of various GSTs (A3, A4, M1, P1 and T1) in order to inactivate aflatoxin B1-8,9-epoxide (AFBO) in alpha mouse liver 12 (AML12) cells [87]. Oral administration of allyl isothiocyanate (AITC) (40 μmol/kg/day) also increased the activity of both quinone reductase (QR) and GSTs in urinary bladder, thus indicating a reduced risk of bladder cancer after frequent exposure to ITCs [88]. Exposure of primary normal human bronchial epithelial cells (NHBE) and lung adenocarcinoma cells (A549) to both broccoli sprout extracts as well as individual ITCs (e.g., SFN, BITC and PEITC) have led to upregulation of GSTP1 (Glutathione S-transferase P) and NQO1 (NAD(P)H dehydrogenase [quinone] 1) phase II enzymes [89]. In addition, SFN, ECN (erucin) and IBN (1-12 μM) were also found to increase the expression levels of thioredoxin reductase 1 (TrxR1) in human breast cancer (MCF-7) cells [90]. Furthermore, SFN also increased the transcription of UDP-glucuronosyl transferase (UGT) 1A1 as well as glutathione S-transferase A1 (GSTA1) in human hepatoma HepG2 and HT29 cells in a time- and dose-dependent manner [91].

#### 1.2.2. Cell Cycle Arrest and Cell Death

The cell cycle consists of four distinct phases: G1, S, G2 and M. Cells go from a resting phase (G0) to proliferation and back to rest. The progression from one phase to the next is mainly regulated by cyclins that form complexes with cyclin-dependent kinases (CDKs) and is inhibited by CDK inhibitors (including p21 and p27) which have a broad affinity for various CDKs. All of these are found deregulated in cancer, thus leading to uncontrolled proliferation of cancer cells [92]. ITCs have been found to induce cell cycle arrest by modulating the expression levels of cell cycle regulators including cyclins and CDKs. Exposure of human colon carcinoma (HT29) cells to SFN (15 μM) was shown to increase the accumulation of these cells in the G2/M phase through activation of *p21* and *cdc2* kinase [93]. A study by Cheng et al., 2016 showed an induced growth arrest in the G2/M phase by inhibiting the expression of Cyclin B1 and disruption of cyclin B1/CDC2 conjugate in cervical cancer cells exposed to SFN (6.25 μM, 12.5 μM and 25 μM) [94]. Alternatively, cell cycle arrest (in G0/G1 phase) and apoptotic induction were observed in T24 bladder cancer cells exposed to SFN (5–20 μM), an effect that was mediated by overexpression of a p27 cyclin-dependent kinase inhibitor [95]. Inhibition of growth (at the G0/G1 phase) through modulation of p53, p21, p17, CDK2 and cyclin E activities was also observed in HSC-3 oral carcinoma cells after exposure to PEITC (0.5, 1, 2, 2.5 and 5 μM) [96]. Cyclin B1, as well as Cdk1, Cdc25B and Cdc25C, expression levels were found to be dramatically decreased in AITC-treated (20 μM) prostate cancer cells with a resultant increase in the number of cells in the G2/M phase [97]. In addition, BITC also induced G2/M phase arrest in human pancreatic cancer (Capan-2) [98] and HL-60 cells [99], at concentrations of 20 μM and 5 μM respectively, by upregulating p21 and also modulating the expression of other regulatory proteins important for cell cycle progression. Moreover, a study by Cheung et al., 2008 assessed the involvement of the p38/MAPK (mitogen-activated protein kinases) pathway in ITC-induced growth suppression. In this work, treatment of HT29 colon cancer cells with an inhibitor specific for *p38* activity attenuated the PEITC-induced (25 μM) G1 arrest, while MAPK activation induced the activity of various other cyclins and, consequently, blocked growth progression [100]. Other studies have also shown that ITCs induced cell cycle arrest in various cancer cell lines, an effect associated mainly with the regulation of the expression levels of p21, GADD45 (growth arrest and DNA damage) and various other cyclins [101,102,103,104]. Finally, only recently, it was proposed that the deregulation of the cell cycle may gradually lead to apoptotic induction [93].

#### 1.2.3. Apoptosis

Apoptosis is a genetically programmed cell death which is characterized by cell shrinkage, chromatin condensation, DNA fragmentation and formation of apoptotic bodies. The apoptotic process is complex and is regulated by a wide range of proteins including (but not limited to) various caspases, B cell lymphoma 2-family (Bcl2) proteins and the *p53* gene. The apoptotic process is deregulated in cancer cells (thus overcoming cell death) and so compounds that can target the induction of molecules involved in the apoptotic process could be of potential benefit as therapeutic agents [105,106]. ITCs have been shown to trigger cell death in cancer cells and their role in apoptotic induction has been excessively studied. The mode of action is complex and it seems that there is a cross-talk between signalling pathways that regulate the transcription of various proteins important for the apoptotic process [62,107,108]. A study by Yu et al., 1998 showed that exposure of human cervical cancer (HeLa) cells to PEITC (and other structurally related ITCs) (10 μM) induced apoptosis, while treatment of these cells with a caspase-3 inhibitor attenuated this phenomenon, thus suggesting that the apoptotic process is strongly linked with caspase-3 activity [109]. To date, many more studies have shown that ITCs exert their anti-proliferative effect through diverse regulatory pathways including: (i) Various signal transduction pathways (PI3K/AKT, MAPKs and mTOR (mammalian target of rapamycin) [110,111], (ii) increased production of reactive oxygen species (ROS) [112], (iii) inhibition of heat-shock proteins [113] and (iv) mitochondrial dysfunction [96,108,114]. In another study, Xu et al., 2006 showed that treatment of prostate cancer (PC-3) cells with SFN (5–40 μM), PEITC (2–10 μM) and AITC (5–100 μM) decreased cell viability by inducing apoptosis. More specifically, ERK1/2 (extracellular signal-regulated kinases), JNK1/2 (c-Jun N-terminal kinases), Elk-1 (ETS-like gene 1, tyrosine kinase) and c-Jun phosphorylation levels were significantly increased, resulting in the induction of activator protein 1 (*AP-1*), which plays an important role in various processes including cell death [115]. The contribution of EGFR (epidermal growth factor receptor)/PI3K (phosphoinositide 3-kinase)/AKT (protein kinase B) and MAPK pathways in ITC-induced apoptosis and cell cycle growth arrest was also documented in a number of other studies, suggesting the importance of these pathways in ITC-induced cytotoxicity [110,116,117,118,119]. On the other hand, modulation in the activity levels of pro-apoptotic (e.g., Bax, Bid and Bak) and anti-apoptotic (e.g., Bcl2 and Bcl-XL) proteins, as well as various other caspases, has been also involved in ITC-induced cell death. Treatment of pancreatic cancer cells with PEITC (2.5 μM, 5 μM and 10 μM) increased protein levels of Bak while decreased those of Bcl2 and Bcl-XL thus inducing apoptosis [120]. In addition, exposure of Jurkat T-leukaemia cells at 30 μM of SFN, resulted in increased Bax and p53 expression levels, which were correlated with an apoptotic induction [121]. Moreover, AITC-induced apoptosis (7.5 μM, 15 μM and 30 μM) was also found to be dependent on elevated levels of Bax together with decreased levels of Bcl2 in renal carcinoma cells [122].

#### 1.2.4. Oxidative Stress

A number of studies also support that ITCs can inhibit cell survival through increased production of reactive oxygen species (ROS). To this end, high concentrations of SFN (10 μM and 20 μM) significantly induced ROS generation in the p53-null osteosarcoma (MG-63) cell line by reducing GSH production and recycling, thus contributing to dysfunction of membrane potential and decreased cell survival. It is noteworthy that, although SFN is a well-known antioxidant agent, under this experimental setting, an imbalance between the antioxidant and prooxidant activities was observed, with the antioxidant activity being dependant on the concentration and exposure period of MG-63 cells to SFN [112]. Also, 10 μM of SFN triggered the intracellular accumulation of ROS, resulting in significantly increased levels of Nrf2 as well as heme oxygenase-1 (HO-1), and consequently, decreased cell survival of bronchial epithelial cells. Pre-treatment with N-acetylcysteine (NAC) suppressed SFN-induced elevation of Nrf2 and HO-1 levels, suggesting that the observed effect was dependent on the oxidative stress response [123]. In line with these observations, exposure of oral cancer cells to BITC, (7.5 μM) and PEITC (10 μM) also caused increased production of oxidative stress leading to GSH depletion, increased expression of serine/threonine protein kinase ATM, Chk2, p53, and p21, while expression of Cdc2, cyclin B1, Mcl-1 and Bcl-2 was suppressed. Thus, ROS elevation resulted in DNA damage, mitochondria disruption and, subsequently, induction of cell cycle growth arrest and apoptosis [124,125].

Another mechanism accounting for ITC-induced inhibition of cell proliferation and survival, through elevation of ROS levels, involves the downregulation of specificity protein (Sp) transcription factors and their gene targets. These transcription factors regulate the expression of various genes involved in proliferation, survival, angiogenesis, inflammation and drug resistance. Overexpression of these proteins is a common finding in multiple cancer types and is associated with poor patient prognosis [126,127,128]. The importance of Sp downregulation has been demonstrated in a recent study where knocking-down Sp1, Sp3 and Sp4 in breast, kidney, pancreatic, lung and colon cancer cell lines reduced cancer cell growth, promoted apoptosis and prevented cell migration and invasion [129]. Recently, BITC and PEITC have been reported to trigger oxidative stress and inhibit proliferation in cancer cells, through a ROS-dependent mechanism that involves the downregulation of Sps [130,131]. These observations are consistent with other studies utilizing ROS-inducing agents with anti-cancer properties such as celastrol [132], HDAC inhibitors [133], penfluridol [134], piperlogumine [135], curcumin and other curcuminoids [136,137], CDDO-Me (a synthetic derivative of triterpenoid glycyrrhetinic acid) [138,139] and GT-094 (a nitric oxide-non-steroidal anti-inflammatory drug) [140]. Their mechanism of action is thought to be ROS-dependent, since their effects are abrogated in the presence of GSH, and involves decreased expression of Sps via modulation of the Myc-miR-ZBTB axis. More specifically, it is proposed that ROS induction inhibits the expression of Myc and Myc-associated micro-RNAs (e.g., miR-27a, miR-20a and miR-17-5p) and, consequently, the activation of zing finger and BTB (ZBTB) inhibitors, thus downregulating Sp transcription factors and their targets [126,141]. Overall, it appears that ITCs can modulate the expression of genes in more than one cellular pathway, important for cell proliferation and survival.

#### 1.2.5. Autophagy

Autophagy is an essential process for cell survival and maintenance through which cells degrade their damaged and defective cytoplasmic components. It occurs when cells are exposed to several stress stimuli, such as low oxygen, starvation and accumulation of faulty organelles and proteins, among others. In general, under ‘housekeeping levels’, autophagy promotes cell survival and growth by removing defective and redundant cellular constituents. However, under conditions of diminished apoptosis or induction of high levels of autophagy, it may also promote cell death, either through self-cannibalization or by inducing apoptosis [142,143]. In the context of carcinogenesis, autophagy can play a dual role either as (i) a tumour-suppressor mechanism by scavenging mutated proteins and faulty organelles (thus inhibiting cell transformation), or (ii) as a tumour-progression mechanism by favouring cancer cell survival and proliferation under hypoxic conditions. Given that autophagy is essential in cellular transformation, the discover of agents capable of inhibiting its induction could be of particular importance in cancer therapy [144,145]. Alternatively, its ectopic activation in cancer cells could also serve as a pro-death signal. To this end, other studies have shown that its induction can inhibit cancer cell growth through activation of T cell-dependent immune responses [146,147].

ITCs can act as autophagic inducers and, by doing so, they may exert either a protective role [148,149,150,151,152,153,154,155,156,157] or promote cell death [158,159,160,161,162] according to tumour type, stage and genetic context. To this end, a recent study investigated the autophagic involvement on BITC-induced inhibition of cell growth in lung cancer cells and showed an induction of ER (endoplasmatic reticulum) stress-mediated autophagy capable of protecting from the suppressive effects of BITC on cancer cell growth. Pre-treatment of cells with an autophagic inhibitor enhanced the growth inhibitory effect of BITC, supporting the cytoprotective role of autophagic induction [149]. In line with these observations, exposure of prostate cancer cells to BITC also triggered autophagy through mTOR signaling inhibition [150] or ROS-accumulation [151]. Inhibition of autophagy in these experimental models found to enhance BITC-induced apoptosis of prostate cancer cells. Moreover, PEITC anti-metastatic potential reported to be significantly increased after inhibition of autophagy and, subsequently, inactivation of the JAK2 (Janus kinase 2)/STAT3 (signal transducer and activator of transcription 3) pathway in three lung cancer cell lines [152]. In contrast, breast cancer cells exposed to BITC are reported to promote autophagic cell death through increased expression of the FOXO1 (forkhead box protein O1) pathway [158]. Also, exposure of prostate cancer cells to PEITC resulted in increased cell death via Atg5-mediated induction of autophagy and apoptosis. In this experimental setting, the apoptotic induction was attenuated after pharmacological inhibition of autophagy [159]. In line, SFN-NAC (sulforaphane-N-acetyl-cysteine), an important metabolite of SFN, was shown to downregulate α-tubulin through induction of autophagy and activation of ERK1/2, leading to cell-cycle arrest and cell growth inhibition in glioma cells [160].

#### 1.2.6. Epigenetic Mechanisms

ITCs can also exert their anti-tumourigenic properties by interfering with the epigenetic machinery [61], which plays an important role in a number of physiological (e.g., genomic imprinting genomic imprinting [163,164,165], X-chromosome inactivation [166,167], development of the embryo and placenta [168,169,170]) as well as disease-modulating processes, including carcinogenesis [171,172,173]. Within the context of carcinogenesis, changes in DNA methylation patterns, as well as post-translational modifications in histone proteins, result in repressed transcription (i.e., silencing) of tumour suppressor genes and/or activation of proto-oncogenes to oncogenes, among other mechanisms [174,175,176].

##### Inhibition of HDACs

Histone deacetylases (HDACs) are generally associated with condensed chromatin conformation leading to transcriptional repression. To date, HDAC inhibitors have been utilized in the clinical setting and are considered to be therapeutic agents against tumour progression by causing cell cycle arrest, inducing cell death and inhibiting angiogenesis [177,178]. To this end, ITCs have been shown to act as potent HDAC inhibitors in various cancer cell lines by inducing genomic alterations resulting in changes in carcinogenic activity of xenobiotics through (i) Nrf2-mediated induction of phase II detoxification enzymes, (ii) induction of cell cycle growth arrest and iii) apoptosis [179,180,181,182]. More specifically, PEITC (10 μM) was shown to cause cell cycle arrest of prostate cancer cells (LNCaP) by enhancing Histone 3 (H3) acetylation which was shown by chromatin immunoprecipitation (ChIP) to be associated with increased expression of p21, thus suggesting the inhibition of HDACs [182]. In addition, SFN (at 15 μM) was also found to be an efficient HDAC inhibitor in BPH-1 (benign prostatic hyperplasia), LnCaP (lymph node carcinoma of the prostate) and PC-3 (prostate epithelial) cells by triggering growth arrest and apoptotic processes [183]. Also, protein expression and activity levels of HDACs 1, 2 and 3 were shown to be significantly decreased in a number of cancer cells exposed to ITCs [184,185,186]. Phenylhexyl ITC (PHI), at concentrations between 1–20 μM, was found to significantly reduce HDAC1 and HDAC 2 activity, in prostate cancer cells, as well as to enhance the acetylation of histones H3, H4 and H3 lysine [186]. SFN, at 15 μM, prevented the repair of DNA damage in human colon cancer (HCT116) cells via diminished HDAC activity levels, accompanied with a marked reduction on the expression of HDACs 3 and 6, resulting in decreased C-terminal-binding protein (CtBP)-interacting protein (CtIP) levels, an important mediator of DNA repair capacity, thus inducing cell cycle arrest and apoptosis [180]. Furthermore, SFN decreased HDAC activity, which led to increased p21 and Bax protein expression levels and induced growth arrest in the G2/M phase and apoptosis in murine melanoma (B16) (concentrations ranging between 3–12 μM), human glioma (U251) (12 μM) [179] and human lung cancer (A549) (15 μM) cells [187]. A study by Jiang et al., 2016 also showed reduction of tumour growth in the lungs of mice exposed to SFN through inhibition of HDAC activity [187]. Other studies also supported the inhibitory effect of ITCs in HDAC activity in both pancreatic [185] and bladder [184] cancer cells. On the other hand, exposure of human pancreatic cancer cells, at 10 μM of BITC, inhibited HDAC1/HDAC3 activity, leading to inactivation of NF-κB and cyclin D1, in vivo and in vitro, thus leading to growth arrest of these cells [185]. Moreover, SFN and erucin (ECN), at 20 μM, attenuated the phosphorylation of Histone 1 isoforms, an important step in bladder cancer development through inactivation of HDACs 1, 2, 4 and 6 [184].

Interestingly, a study by Myzac et al., 2004 was the first to report on the inhibitory effect of SFN on HDACs [188]. In fact, in this study, it was documented that two conjugated compounds of SFN metabolism [SFN-Cys (cysteine)and SFN-NAC (N-acetylcysteine conjugate)] were responsible for the inhibition of HDAC activity and not the parent compound itself. This observation was confirmed by pre-treating cells with a GST inhibitor, thus impairing the first step of the mercapturic acid metabolic pathway of SFN and consequently the production of its major metabolites. Also, treatment of cells with SFN, SFN-GSH, SFN-Cys or SFN-NAC showed that only the two metabolites could effectively inhibit HDACs, while SFN and SFN-GSH had no significant inhibitory activity in vitro [188]. Between the two metabolites, SFN-Cys was found to be the more potent inhibitor. In fact, the order of such inhibition efficiency was documented to be as follows: SFN = SFN–GSH < SFN–NAC < SFN–Cys. Furthermore, by utilizing molecular modeling methodologies, it was revealed that SFN–Cys’s inhibitory capacity was via direct interaction with HDACs, suggesting its potential role as a competitive inhibitor. To this end, it was shown that the α-amino group of SFN–Cys fits into the enzyme pocket and its carboxylate group creates a ligand with the zinc atom within the active site of the ezyme [188,189]. These insights into how SFN-conjugates bind and consequently suppress HDACs have led to the identification of other structurally related ITCs like sulforaphene, phenylbutyl isothiocyanate, phenethyl isothiocyanate and erucin, among others [189,190].

##### Inhibition of DNMTs

DNA methylation results in the addition of methyl groups in cytosine residues of CpG dinucleotide regions that are dispersed across the genome [191,192]. The transfer of these methyl groups is utilized by DNA methyltransferases (DNMTs) with the most important ones being DNMTs 1, 3A and 3B [193,194]. Both hypo- and hyper-methylation have been observed to enhance the expression of oncogenes while inhibiting that of tumour suppressor genes, respectively [195]. ITCs have been shown to cause changes in DNMT expression patterns, thus leading to differential expression of genes capable of mediating the carcinogenic process [196].

In prostate cancer cells, SFN suppressed the expression of DNMT1 and DNMT3A thus decreasing cell proliferation [197,198]. To this end, it has been proposed that SFN-induced inhibition of DNMT1 is associated with HDAC suppression, which can lead to decreased histone deacetylation leading to re-expression of *p21* and impairment of DNMT1 activation [196]. Phenylhexyl isothiocyanate (PHI) (ranging between 5 and 40 μM) was also found to reduce the activity of DNMT1 and 3B (thus causing the re-expression of the p15 tumour suppressor gene), in addition to causing an increased acetylation of histones H3 and H4 in leukaemic T cells [199]. In general, it has been demonstrated that ITCs are epigenetic agents with dual activity on the epigenome, by inhibiting both DNA hyper-methylation and histone deacetylation [181,200,201]. To this end, SFN at 2.5 μM and 5 μM has been shown to induce Nrf2-dependent inhibition of skin tumour transformation in mouse JB6 cells by decreasing the expression of DNMT1, DNMT3A and DNMT3B, as well as that of HDACs 1, 2, 3 and 4 [181]. Similarly, SFN at 1 μM and 2.5 μM, decreased Nrf2 promoter methylation and histone deacetylation, both events leading to an increase in N-ribosyldihydronicotinamide quinone reductase (NQO)-1 transcription in prostate cancer cells [200]. SFN-treated human breast cancer cells (5 μM and 10 μM) underwent apoptosis mediated by downregulating the expression and activity of DNMT1 and DNMT3A, thereby repressing the activation of the human telomerase reverse transcriptase (*hTERT*) gene commonly overexpressed in many cancers [202]. On the other hand, in estrogen receptor (ER)-negative breast cancer cells exposed to 10 μM of SFN-suppressed protein, expression levels of HDACs and DNMTs both contributed to decreased cell viability, suggesting its usage as a therapeutic agent for the treatment of patients with ER-negative breast cancer [201]. Finally, Ras-association domain family 1 isoform A (*RASSF1A*) is a tumour suppressor gene found downregulated in a number of cancers. Exposure to 5 μM PEITC restored *RASSF1A* expression levels and decreased viability levels by inhibiting the activity levels of DNMT1 DNMT3A and DNMT3B, as well as those of HDACs 1, 2, 4 and 6 [203].

##### Modulation of miRNAs

mi-RNAs are functional non-coding RNAs 20–24 nucleotides long that play a critical role in regulating various physiological processes; they are also implicated in disease development, including cancer. Various mi-RNAs such as miR-155 and miR-21 are deregulated during carcinogenesis, promoting tumour development and progression [204]. Many studies have shown that ITCs exert their effect by regulating the epigenetic machinery through targeting of specific miRNAs in cancer. For instance, when epithelial colon (NCM460 and NCM356) cell lines were exposed to 10 μM SFN and IBN, upregulation of miR-23b, miR-27b (tumour suppressors) together with downregulation of miR-155 were evident, thus suggesting their contribution in chemo-prevention [205]. Prostate cancer (PC3) cells exposed to 2.5 μM PEITC overexpress miR-194, which decreases expression of matrix metalloproteinases (MMPs) and blocks the invasion capacity of these cells [206]. In non-small cell lung cancer (NSCLC), miR-616-5p is overexpressed and, consequently, promotes tumour progression and metastasis. In this experimental setting, exposure to 5 μM SFN inhibits growth and migration of non-small cell lung cancer cells by downregulating miR-616-5p [207]. Moreover, combinational treatment of SFN with temozolomide (TMZ) enhanced TMZ’s potential for apoptotic induction by suppressing miR-21 expression [208]. In bladder cancer cells, epithelial-to-mesenchymal transition (EMT) inhibition by SFN (2.5 μM, 5 μM, 10 μM) was partly due to the upregulation of miR-200c, resulting in the suppression of Cyclooxygenase (COX-2) and MMP2 and MMP9, which are important for cellular migration [209]. Finally, in prostate cancer, androgen receptor (*AR*) activity is important for initiation and progression. In this context, PEITC (10–20 μM) blocked AR transcriptional activity by decreasing miR-141 together with increasing miR-17 expression, followed also by an induction of small heterodimer partner (shp) activity (a repressor of AR) and suppression of p300/CBP (CREB-binding protein)-associated factor (PCAF) (co-regulator of AR) respectively [210,211].

#### 1.2.7. Anti-Angiogenic and Anti-Metastatic Properties

New vascularization is an important step in tumour progression by supporting increasing demands for nutrients and oxygen and thus contributes to growth, invasion and metastasis [212]. Metastasis is a complex process, characterized by the loss of cellular adhesion, increased invasiveness, entry to circulation, colonization and proliferation into new tissue. The metastatic process is mainly mediated by genes that play an important role in the modification in the interaction of cells with extracellular matrix and angiogenesis. Among them, MMPs are of significant importance by mediating the extracellular matrix (ECM) degradation, thus promoting cell migration and angiogenesis. Although many MMPs are found overexpressed during carcinogenesis and angiogenesis, MMP-2 and -9 are the most extensively characterized ones [213]. On the other hand, angiogenesis occurs in six sequential steps, i.e., vascular destabilization, extracellular matrix degradation, migration and proliferation of endothelial cells, tube formation and vascular stabilization. The process is regulated by a number of molecules that include MMPs, plasminogen activators (PAs) and integrins among others. The most studied pro-angiogenic factor is vascular endothelial growth factor (VEGF). An imbalance between angiogenic factors and angiogenesis inhibitors play a pivotal role in carcinogenesis [214,215].

Many studies have tried to elucidate the mechanism of the anti-angiogenic and anti-metastatic capacity of ITCs. In general, it has been proposed that ITCs regulate the expression of important genes that control a wide range of cellular processes, including cell cycle arrest, cell death, angiogenesis, cell migration, etc., through modulation of various signalling cascades like p38/MAPK and mTOR pathways [216,217,218]. To these ends, exposure of human umbilical vein endothelial (HUVECs) cells to 5 μg/mL AITC and PITC downregulated VEGF and various pro-inflammatory cytokines (e.g., IL-1β, IL-6, TNF-alpha), while upregulating IL-2 and tissue inhibitor of metalloproteinases (TIMP), thus preventing migration, invasion and tube formation [218]. Similarly, 4 μM PEITC was found to suppress the secretion of VEGF, downregulate VEGF receptor 2 protein levels and inactivate the prosurvival serine–threonine kinase Akt, thus preventing new vascularization in HUVECs [219]. BITC, at 2 μM and 5 μM, was also shown to be effective in inhibiting new capillary formation and invasion of human glioma cells as well as causing G2/M arrest by regulating the expression of molecules important for cell cycle progression (cyclin B1 and p21) and the neo-angiogenetic process (MMPs-2 and -9 and vascular endothelial cadherin (VE-cadherin). Furthermore, modulation in the expression of tumour suppressor (miR-144, miR-122) as well as oncogenic (miR-181b, miR-9) mi-RNAs also was observed following the BITC treatment, suggesting involvement of post-translational modifications in the anti-angiogenic properties of BITC [216].

Hypoxia promotes tumour progression, maintenance and metastasis by stimulating angiogenesis. Thus, hypoxia-induced angiogenesis is related to hypoxia inducible factor-1α (HIF-1α) expression, which in turn stimulates VEGF-induced vascular remodelling and is partially regulated by the mTOR signalling pathway [214,220]. In an experimental setting of hypoxia-exposed human colon and gastric cancer cells, exposure to SFN, at concentrations ranging between 12.5 and 50 μM, dramatically decreased HIF-1α and VEGF protein levels, as well as hypoxia-induced migration [221]. Similar results were observed when breast (6.5–26 μM) and renal (5–40 μM) cancer cells were exposed to PEITC under hypoxic conditions (i.e., suppressing HIF-1α and VEGF protein levels) [222]. In turn, VEGF inhibition leads to a decreased expression profile of other proteins also associated with the vascularisation process. Overall, PEITC-induced inhibition of angiogenesis was found to be PI3K/AKT and ERK/MAPK dependent suggesting an important role of these signalling pathways in the angiogenetic process [223].

The epithelial-to-mesenchymal transition (EMT) is a developmental process of epithelial cell transformation characterized by loss of polarity and adhesion and an enhancement of migratory and invasive properties. The process is regulated by various molecules (e.g., E-cadherin, vimentin, MMPs) and pathways (e.g., Wnt and Notch) [224,225]. Inhibition of EMT by SFN, in thyroid cancer cells, has been associated with modulations in the activity of major signalling pathways (PI3K/Akt and p38/ERK/JNK/MAPK) and depends on ROS production in vitro (20–40 μM) and in vivo (50 mg/kg). Molecules that contribute to EMT process such as E-cadherin and MMP-2 and -9 were also downregulated after exposure to SFN [226]. BITC also was able to repress EMT partially through Forkhead Box Q1 (FOXQ1) suppression in both breast cancer cells (2.5 μM and 5 μM) and xenografts (7.5 μmol BITC/mouse), thus reducing their metastatic potential [227]. Finally, another study indicated that VEGF suppression was mediated through inhibition of the Forkhead Box O1 (FOXO1)/AKT pathway as well [228]. In another set of experiments, the anti-metastatic potential of AITC (5 μM and 10 μM) was evident after inactivation of the MAPK signalling pathway and downregulation of MMPs-2 and -9 in human colorectal adenocarcinoma (HT-29) cells [217]. Furthermore, exposure of these cells to 0.01–10 μM BITC inhibited their growth in a dose-dependent manner and altered their metastatic potential by reducing the expression levels of MMP2 and MMP9 and (urokinase-type plasminogen activator (u-PA) [229]. On the other hand, 10–15 μM SFN was also shown to act as an anti-angiogenic agent through its capacity to supress endothelial cell proliferation, impair cellular microtubule polymerization and decrease the expression levels of various metastatic markers (e.g., HIF1α, MMP-2, VEGF) in human endothelial cells [230,231]. In vivo studies, utilizing Balb/C mice implanted with VEGF-impregnated matrigel plugs, further support the anti-angiogenic activity of SFN (100 nmol/day, intravenously for seven days) since it decreased VEGF-induced vascularization [230].

## 2. ITCs as Cancer Chemo-Preventive and Chemo-Therapeutic Agents

### 2.1. ITCs as Cancer Chemo-Preventive Agents: Human Studies

Over the last decades, ITCs have raised scientific interest as agents capable of preventing cancer development [63,232,233,234,235,236,237], though such protective effects might depend on the individual’s genetic variation in (glutathione S –transferase) GST genes, given their important role in the metabolism and excretion of ITCs as well as in the inactivation of xenobiotics [238,239,240]. Thus, a reduced activity of these enzymes is associated with a longer exposure of tissues to ITCs, which significantly decreases the risk of disease. Therefore, people with inherited two null variants of the glutathione S-transferase Mu 1 (*GSTM1*) and glutathione S-transferase theta 1 (*GSTT1*) alleles are more resistant to cancer development due to longer exposure to ITCs [241,242,243]. However, current studies support the association between GST-null polymorphism with an increased risk of cancer [244,245,246] while others suggest that there is no association between cancer risk and GSTs polymorphisms [247,248]. Consequently, it is obvious that the influence of GST genotype on cancer susceptibility is still ill defined as a result of many inconsistent and contradictory studies in this field of research.

However, epidemiological evidence supports the hypothesis that a diet high in cruciferous vegetables can gradually reduce the risk of cancer development. An inverse correlation between cruciferous vegetables consumption and risk of pancreatic cancer was reported in a case-control study that included 390 prostate cancer cases and 414 controls in urban Shanghai [249]. In another case-control study, a protective effect of cruciferous vegetable consumption was documented against colorectal cancer risk in women from Shanghai (322 cases and 1251 controls), which was associated with *GSTM1*- and *GSTT1*-null polymorphisms [250]. A study by Zhao et al., 2007 also showed that a diet rich in cruciferous vegetables decreased the risk of bladder cancer development. In this case-control study, 694 newly diagnosed patients and 708 controls participated and found that the highest consumption of cruciferous vegetables was related to a 29% decreased risk for bladder cancer development [239]. Furthermore, in another case-control study of 1619 prostate cancer patients and 1618 controls (African-Americans, Caucasian, Japanese and Chinese men), it was shown that an inverse association exists between cruciferous vegetable consumption and prostate cancer development [251]. Other case-control studies have shown that higher dietary intakes of cruciferous vegetables can decrease the risk of lung cancer, especially squamous and small cell lung carcinomas [252]. For instance, a case-control study of 420 Chinese women showed that a diet rich in ITCs led to a decreased rate of lung cancer incidence, especially in smokers [243]. Moreover, a 32% reduction in the risk of lung cancer was observed in individuals who consumed three or more weekly servings of cruciferous vegetables compared to those consuming one-half servings or less (meta-analysis of β-carotene and retinol efficacy trial (CARET) trial conducted in 14,120 participants) [253]. Several other case-control studies also support a beneficial effect of dietary ITCs in breast cancer. More specifically, studies in Caucasian (740 cases and 810 controls) and Chinese (337 cases and 337 controls) women found that cruciferous vegetables intake was significantly lower in women diagnosed with breast cancer than women in control groups [254,255]. Finally, there are several more case-control studies associated with low intake of cruciferous vegetables and higher risk of developing various types of cancer [256,257,258]. On the other hand, no significant association was observed between cruciferous vegetable consumption and risk for liver cancer in a study of 217 patients and 42 corresponding controls in Shanghai [259]. In addition, no significant association was reported between low cruciferous vegetable consumption and lung cancer susceptibility among smoking women (209 incident lung cancer cases and 787 individually matched non-smoking controls) although a meta-analysis showed an association between urinary ITC levels, GSTM1 genotype and lung cancer risk [260]. These observations contradict with previous studies, although the majority of research outcomes suggest that there is an inverse relationship between intake of ITCs derived from cruciferous vegetables and the overall incidence for various types of cancer development.

### 2.2. ITCs as Cancer Chemo-Therapeutic Agents: Animal Studies

#### 2.2.1. In Vitro Studies Utilizing Various ITCs

In a human breast adenocarcinoma cell model, exposure of cells to AITC (5, 10, 15 and 20 μM) induced generation of oxidative stress as well as the ERK signalling pathway, both of which resulted in activation of apoptosis (e.g., increased expression of caspase-3 and -9), growth arrest of cells in the G2/M phase (e.g., increased expression of p21 and suppression of cyclin B and CDK1), mitochondrial depolarization and deregulation of mitochondrial-associated proteins (e.g., decreased expression of Bcl-2 and increased expression of cytochrome c and Apaf-1) [111]. In line with these observations, a study by Wu et al., 2011 reported elevated ROS levels that led to an increase in nitric oxide (NO) production, deregulation of mitochondria potential, cell cycle suppression and apoptosis in osteogenic sarcoma cells exposed to BITC (7.5 μM) and PEITC (10 μM) [261]. In a breast cancer cell model consisting of MDA-MB-231 and MCF-7 cells, exposure of cells to BITC (2.5–20 μM) resulted in a significant decrease of cell viability mediated by ROS production, mitochondrial dysfunction, deregulation of pro- and anti-apoptotic genes and activation of various caspases [262]. In addition, PEITC (0.5–5 μM) was found to be an effective inhibitor of oral squamous carcinoma cell growth through cell cycle arrest and mitochondrial-dependent apoptosis due to ROS production and Ca^2+^ accumulation [96]. On the other hand, AITC (1–40 μM) induced a dose-dependent inhibition of the survival of human A549 and H1299 non-small cell lung cancer (NSCLC) cells by inducing replication stress as well as sensitizing tumour cells to radiation [263]. Moreover, it has been shown that even low doses of PEITC (0.1–10 μM) were able to block cell growth and proliferation of prostate cancer (LNCaP) cells [264]. Finally, exposure of human colon cancer cell lines to SFN and PEITC (0.1–100 μM) have led to a dose-dependent inhibition of their proliferation as well as apoptotic induction [265].

Exposure to PHI (5–40 μM) blocked cell cycle progression in human leukaemia cells through perturbations in acetylated as well as methylated states of chromatin histones [266]. In comparison, in human embryonic kidney 293 cells and human colorectal cancer (HCT116) cells, exposure to SFN (15 μM) caused a decrease in HDAC-3 and -6 activity levels while raising p21 expression levels suggesting that SFN could act as an effective tumour-suppressor agent [188,267]. SFN (15 μM) was also found to be an efficient HDAC inhibitor in BPH-1, LnCaP and PC-3 prostate epithelial cells resulting in triggering of growth arrest and induction of the apoptotic process as well [183]. Furthermore, in prostate cancer, *GSTP1* methylation is known to play an important role in tumour initiation. In this context, PEITC (0.5–20 μM) was found to suppress the deacetylation and methylation of the *GSTP1* gene and thus block the carcinogenic process [268].

Exposure of prostate cancer cells to PEITC (5 μM and 7.5 μM) and SFN (20 μM and 30 μM) significantly inhibited phosphorylation of IKK/IκB kinases and p65 as well as NFκB subunit nuclear translocation, thus suppressing the expression of NFκB-related genes (e.g., VEGF, cylcin D1 and B-cell lymphoma-extra large (Bcl-XL) leading to disturbing angiogenesis and invasion [269]. Alternatively, the signal transducer and activator of transcription 3 (STAT3) factor is found to be overexpressed in various cancers where it favours tumour growth and progression. A study by Boreddy et al., 2011 has shown that BITC (5–20 μM) inhibited the phosphorylation of STAT3 in pancreatic cancer cell lines followed by a decrease in *VEGF* and MMP-2 expression, thus blocking angiogenesis [270].

In addition, it was proposed that ITCs protect against tumourigenesis by enhancing the ubiquitination process of oncogenes, thus favouring their degradation by the proteasome. In fact, both BITC and PEITC have been shown to target USP9x (ubiquitin specific peptidase 9 X-linked), a member of deubiquitinating enzymes (DUB), thus promoting the anti-apoptotic protein Mcl-1 (myeloid cell leukaemia-1) and the oncogenic fusion protein Bcr-Abl for degradation in various tumourigenic cell lines [271]. Finally, another target of ITCs’ anti-proliferative effect is tubulin, which is known to disrupt microtubule polymerization and, consequently, induce mitotic arrest and apoptosis [272,273,274]. Thus, it becomes evident that a number of studies support the plurality of effects of ITCs in various cancers including ovarian [275], glioma [276], bladder [277], breast [278,279], myeloma [280], prostate [281,282,283] and colon [284].

#### 2.2.2. In Vivo Studies Utilizing Various ITCs

Oral administration of AITC in the form of mustard seed powder (9 μmol/kg or 90 μmol/kg body weight, once daily for three weeks) significantly prevented tumour development in mouse bladders and invasion in muscles by triggering cell cycle arrest and apoptosis while inhibiting angiogenesis [285]. At the same time, BITC (5 mg/kg and 10 mg/kg body weight, every day for 19 weeks) suppressed the development of prostate cancer in a transgenic mouse adenocarcinoma model by inducing cell cycle arrest. Moreover, BITC resulted in reduced Ki67 (a proliferation marker), cyclin A, cyclin D1 and CDK2 expression in the prostatic tissue [286]. A study by Srivastava et al., 2003 also demonstrated that injection of 10 μM AITC three times per week significantly inhibited the growth of PC-3 xenografts in vivo by inducing apoptosis, thereby decreasing the proportion of cells undergoing mitosis [287]. SFN (at an average daily dose of 7.5 μmol per animal for 21 days) also decreased prostate cancer cell proliferation in xenografts by suppressing HDAC activity. A reduction in HDAC activity was also observed in the peripheral blood mononuclear cells of healthy humans after consumption of broccoli sprouts (a single dose of 68 g broccoli sprouts, approximately 105 mg SFN; equivalent to approximately 570 g of mature broccoli) [288]. Intraperitoneal administration of SFN in nude mice (25 mg/kg, 50 mg/kg or 100 mg/kg, 3 times/week) injected with lung adenocarcinoma (LTEP-A2) cells significantly decreased cell proliferation and thus tumour progression [289]. Moreover, 5 μM SFN and PEITC found to significantly reduce the formation of colonic aberrant and multicrypt foci in F344 rats [290]. Also, mice inoculated with pancreatic cancer (BxPC-3) cells and then orally treated with 12 μM BITC showed 43% less tumour growth (in comparison to control mice) through repression of PI3K, AKT, PDK1, mTOR, FOXO1 and FOXO3a, in addition to an increased apoptotic induction [291].

On another note, a study by Xu et al., 2006 also described that topical application of 100 nmol of SFN once a day was capable of suppressing 7,12-dimethylbenz(a)anthracene (DMBA)/12-O-tetradecanoylphorbol-13-acetate (TPA)-induced skin tumourigenesis, in C57BL/6 mice, an effect which was mediated by Nrf2 [292]. Microarray-based identification of gene expression patterns in the liver of both wild-type and Nrf2 knock-out C57BL/6J mice exposed to SFN (90 mg/kg administered orally) revealed that SFN induced Nrf2 activity. This, in turn, regulates the expression of various other proteins, like phase II detoxifying enzymes (e.g., HO-1, GSTs), cell cycle and growth associated proteins (cyclins, growth factors), heat-shock proteins (e.g., Hsp90) and various kinases (e.g., PI3K, MEKs) thus confirming previous observations on ITC-induced Nrf2 activity [293]. PEITC (12 μmol/day, 5 days/week) was also found to inhibit tumour formation in human pancreatic cancer (MIAPaca2) cell line xenograft mice [120]. In bladder cancer, both SFN and ECN (295 μmol/kg orally once a day) were found to significantly block HDAC activation in vivo [184].

Finally, the anti-angiogenic and anti-metastatic effects of ITCs have been also demonstrated in vivo. More specifically, oral administration of 12 μM BITC suppressed new vessel formation and suppressed pancreatic tumour growth in mice through inhibition of STAT3 phosphorylation. The tumours collected from mice were characterized by decreased expression of angiogenesis-related proteins, including HIF-α, VEGF, MMP-2 [270]. On the other hand, oral administration of 10 μM PEITC in MDA-MB-231-BR xenografts prevented the growth of tumour and enhanced the survival of xenografts compared to controls, while the expression levels of VEGF were downregulated in the tumour-bearing mice [294]. In addition, oral administration of 50 μmol/kg or 150 μmol/kg of PEITC inhibited capillary formation and attenuated tumour invasiveness in a chemically induced murine breast cancer model [295].

## 3. ITCs as Anti-Melanoma Agents

Malignant melanoma is a highly aggressive and metastatic type of skin cancer with adverse prognosis, high mortality rates and a poor response to current therapeutic strategies. Its incidence is multifactorial. The most important risk factor for melanoma development is exposure to UV (ultraviolet) radiation by means of prolonged exposure to solar energy and/or frequent use of tanning beds. Other factors that increase the likelihood of disease development include innate predisposition and inheritable traits including fair skin, sun sensitivity and/or a large number of moles [296]. The aggressive nature of melanoma is associated with the accumulation of mutations that activate oncogenes (e.g., *BRAF*, *NRAS*), while inactivating tumour suppressor genes (e.g., phosphatase and tensin homolog; *PTEN)* [297]. Overall, the molecular pathways that regulate the expression of these genes are constituently activated in melanoma. More specifically, the most important pathways found to be overexpressed include p38/JNK/ERK/MAPK, PI3Kinase/Akt/mTor, β-catenin and Wnt. Deregulation of proteins important for cell cycle progression and apoptosis also account for the increased survival and proliferation of melanoma cells. Anti-apoptotic proteins such as Bcl-2 and Bcl-xL are overexpressed, while pro-apoptotic modulators such as Bax, Apaf-1 and p53 are suppressed in melanoma. Angiogenesis also plays an important role in melanoma progression and metastasis, thus molecules capable of targeting genes related to both of these processes may be part of an effective anti-melanoma strategy [298,299]. Finally, epigenetic modifications in pathways controlling cell growth, proliferation, motility and apoptosis have also recently been identified in malignant melanoma cells [300].

The anti-carcinogenic effect of ITCs in malignant melanoma has been studied by utilizing both in vitro and in vivo models. In general, ITCs were found to be able to induce growth arrest and apoptosis [301] and also inhibit metastasis [302,303] through induction of various signal transduction pathways, generation of oxidative stress and disruption of mitochondrial function, all of which are important mediators of cell growth and proliferation [296].

### 3.1. In Vitro Studies Utilizing Various ITCs

The anti-melanoma effect of ITCs has been studied by utilizing in vitro models of both human and murine species. In murine (B16F-10) melanoma cells, activation of multiple caspases, pro- and anti-apoptotic proteins and pro-inflammatory cytokines (e.g., TNF-α, interleukin-1β, IL-6, IL-12p40 and NF-κB) were all shown to be involved in SFN-induced apoptosis (1–5 μM) [304]. Furthermore, exposure of these cells to 1–5 μg/mL AITC and PITC depleted the expression and secretion of agents that favour blood vessel formation (e.g., TNF-α and NO) [305]. Such anti-angiogenic capacity was also exerted by AITC and PITC in B16F-10 melanoma cells through decreased expression of VEGF at 5 μg/mL [216]. Moreover, the expression of platelet-activating factor receptor (PAF-R) is linked to a higher metastatic potential in melanoma cells. Exposure to 5–50 μM BITC significantly increased apoptotic cell death in murine PAF-R positive-cells in contrast to PAF-R negative B16F10 cells, suggesting selectivity of BITC towards PAF-R, which is promising for a better management of PAF-R-positive melanoma patients. The apoptotic process was found to be mediated by elevated ROS production and caspase3/7-like activity [306]. In line, another study by Lai et al., 2017 also supported the anti-metastatic potential of BITC and PEITC (1 μM, 2.5 μM and 5 μM) in B16F-10 cells by showing that both ITCs decreased cell viability, mobility, migration and invasion via inhibition of MMP-2 activity and impaired expression of important metastasis-related proteins like MAPK signalling-associated proteins (e.g., p-ERK1/2, p-p38 and p-JNK1/2), RhoA, Ras, SOS-1, FAK, GRB2, TIMP and NF-κB [307]. Similarly, BITC (0.5 μM, 1 μM and 2 μM) and PEITC (1 μM, 2 μM and 2.5 μM) also prevented human melanoma A375.S2 cell migration and invasion through inhibition of MMP-2 activity as well as via affecting the MAPK signalling pathway [308]. Treatment with SFN at 20 μM has been shown to decrease cell survival through activation of p38 and p53, which regulate the expression of pro-apoptotic proteins, such as Bak and PUMA, in both Bowes and SK-Mel-28 human melanoma cells [309]. In addition, exposure of human malignant melanoma (A375) cells to 0.1–5 μM SFN, BITC and PEITC exerted a cytotoxic effect via multiple apoptotic pathways (intrinsic, extrinsic and endoplasmic reticulum-based), as shown by the increased activity of various caspases indicative of such differential activation [310]. A study by Huang et al., 2012 also showed that addition of PEITC at concentrations of 5–15 μM inhibited the growth of A375.S2 cells by causing G2/M-dependent cell cycle arrest and inducing the intrinsic apoptotic pathway through ROS-mediated mitochondrial dysfunction [301]. Similarly, exposure of A375.S2 melanoma cells to 5–20 μM BITC decreased cell growth and survival in a dose-dependent manner. BITC promoted ROS accumulation in cells and caused changes in the expression of various cyclins and CDKs (e.g., cyclin A, CDK1, CDC25), proteins of the B-cell lymphoma 2 (BCL2) family and various caspases [311]. In line with these observations, 5 μM of SFN, BITC and PEITC have also been documented to induce G2/M cell cycle arrest by affecting the expression levels of various cell-cycle regulators in human malignant melanoma (A375) cells [312]. Moreover, the effect of AITC on lysine acetylation and methylation marks on histones H3 and H4 have been explored recently. To this end, exposure of A375 cells to 10 μM of AITC decreased HDAC and histone acetyl transferase (HAT) activities, while also affecting the acetylated and methylated content of histones (by means of assessing specific lysine modifications), suggesting that AITC can exert an epigenetically-induced anti-melanoma activity [313].

ITCs were also found to affect TRPA (transient receptor potential) ion channels in melanoma cells, which are responsible for calcium efflux in various types of skin cells and are involved in both physiological (e.g., epidermal homeostasis, sensory function) and pathological (e.g., melanoma) processes. For example, although TRPM1 (transient receptor potential cation channel subfamily M member 1) is expressed in melanocytes mediating melanin production in melanoma, TRPA1 promotes tumour progression and invasiveness and is linked to a more aggressive disease phenotype [314]. Furthermore, TRPA1 was found to be functionally expressed in various human malignant melanoma cell lines but, at the same time, was not critical for an impaired proliferation caused after exposure of these cells to AITC (25–400 μM) [315].

On the other hand, oil extracted from the roots and seeds of the plant *Eruca sativa* (rocket salad; a member of Cruciferous family) has been reported to exert an anti-melanoma activity. Exposure of B16F10 murine and MDA-MB-435 human melanoma cell lines to 20–100 μg/mL of the extract (major components were ECN, SFN, AITC, 3-butenyl-ITC and 2-phenylethyl-ITC) significantly inhibited cell proliferation [316]. Also, combinational treatment of AITC, PEITC and SFN (3–50 μM) was found to be a more potent inhibitor of B16F-10 proliferation than the seed oil extract itself [317]. Finally, ITCs decreased proliferation in melanoma stem cells (MSCs), a subgroup of malignant melanoma cells with high metastatic and invasive capacity. MSCs express Ezh2 polycomb group protein that plays an important role in cell survival and which was shown to be suppressed after exposure to 1–20 μM SFN, thereby reducing cell viability, migration and invasion in these cells [318]. However, it was recently reported that the various biological effects of SFN can be attenuated from the presence of other biological factors, in vivo (e.g., the nerve growth factor, etc.), suggesting that a better understanding in the interaction of these compounds with other elements in the body is crucial in order to determine their in vivo efficacy [319].

### 3.2. In Vivo Studies Utilizing Various ITCs

The cytotoxic effect of ITCs has been shown in a number of malignant melanoma animal models. To this end, a study by Bansal et al., 2015 documented that a combinational treatment consisting of allyl isothiocyanate, phenylethyl isothiocyanate and sulphoraphane (at 1:1:1 ratio; 10 μM) decreased tumour growth, volume and weight in C57BL/6 mice injected with B16F-10 melanoma cells and that such a combination was more potent than treatment with naturally occurring *Eruca sativa* seed oil alone [320]. Intraperitoneal injection of 20 mg/kg and 40 mg/kg PEITC as well as 20 mg/kg BITC significantly decreased the size and weight of tumours in BALB/c mice injected with A375.S2 cells, suggesting that both agents can be effective in melanoma chemotherapy [321,322]. The anti-metastatic potential of both AITC and PEITC has been shown to be linked with inhibition of angiogenesis. More specifically, in C57BL/6 mice inoculated with B16F-10 melanoma cells, capillary formation was prevented after exposure to AITC and PITC (25 μg/dose/animal/day), an effect which was mediated by reduction in the expression levels of NO and TNF-α, both of which promote capillary formation [305]. In another study, exposure to AITC and PITC intraperitoneally at a dose of 1.1 mg/kg also inhibited the B16F-10-induced metastasis in the lungs of C57BL/6 mice [323]. In line with these observations, SFN (administered intraperitoneally at a dose of 500 μg/dose/animal/day) also blocked the metastatic potential of B16F-10 cells inoculated in C57BL/6 by inducing a cell-mediated immune response and triggering the expression of IL-2 and IFN-gamma, while suppressing those of IL-1beta, IL-6, TNF-alpha and GM-CSF (granulocyte-macrophage colony-stimulating factor) [324]. Moreover, SFN (3.5 mg/kg body weight, injected thrice a week) inhibited cell proliferation and migration in B16F10 xenografts, effects which were both mediated by a reduction in the expression levels of MMP-9 [325]. Additionally, ITCs were also shown to play a role in melanoma epigenetic therapy. More specifically, SFN inhibited growth and proliferation of B16 and S91 murine melanoma cells by downregulating deacetylation enzymes and intraperitoneal injections of SFN-encapsulated microspheres (500 μmol/kg) enhanced its anti-cancer activity in melanoma tumour-bearing C57BL/6 mice [326]. Also, the effect of SFN in Ezh2 stem cell survival protein was assessed after oral administration in mice inoculated with A375 melanoma cancer stem cell (MCS) cells. SFN (10 μmol/kg body weight; three times per week) inhibited tumour growth, an effect which was associated with reduced expression levels of matrix metalloproteinases, increased expression levels of TIMP3 (Metalloproteinase inhibitor 3) and enhanced apoptosis [318]. Based on our current knowledge of ITCs’ effectiveness in melanoma prevention, synthetic analogues (with longer side chains with selenium substituting the sulphur group) have been produced and shown to be effective in inhibiting melanoma survival by regulating signalling pathways like the Akt3 pathway, but without affecting normal cells [327,328,329]. For instance, naphthalimide is one such ITC synthetic analogue that has been found to exert cytotoxicity in both in vivo and in vitro experimental settings [330].

## 4. Conclusions

ITCs are potent electrophiles derived from the breakdown process of GLs, which are abundant in cruciferous vegetables. They are currently considered as an important class of nutraceuticals, characterized by a wide range of properties (e.g., anti-bacterial, anti-inflammatory, anti-cancer) suggesting their possible use in various industries ranging from food to medicine to clinical practice. Their potential application in cancer prevention strategies have posed greater interest over the last years. According to various studies, the consumption of cruciferous vegetables has been associated with a reduced risk of cancer development, thus supporting their protective role. In addition to their protective role, a large body of evidence shows the anti-tumourigenic action of ITCs against different types of cancer both in vitro and in vivo. The observed tumour-inhibitory effects of ITCs have been shown to be mediated through modulation of various cancer-related critical pathways (e.g., enzymatic detoxification, apoptotic induction, oxidative stress generation, signal transduction, epigenetic induction, etc.) and they seem to be more potent against cancer than normal cells. The most important and well characterized pathway associated with the protective effect of ITCs is the Nrf2 pathway, which is associated with the detoxification and elimination of carcinogens from the body. However, various studies have shown its constitutive expression could lead to resistance in chemotherapy. Despite that fact, the overwhelming majority of studies support the protective role of these agents which (together with their low toxicity profile) makes them excellent chemotherapeutic candidates against tumour initiation and progression. However, further studies are needed in order to determine the optimum dose-response and time-course of ITCs, as these parameters can be a significant limitation for their long-term usage in cancer therapeutic interventions.

Melanoma is a highly aggressive type of skin cancer being linked with high mortality rates due to its high metastatic potential and treatment resistance. As such, the disease does not always respond to current therapeutic approaches, largely due to the development of multi-drug resistance. Thus, it is imperative that new potent chemotherapeutic agents are developed for disease management. To this end, ITCs have been studied for their anti-melanoma efficacy by means of interfering with various pathways including inhibition of cell growth and proliferation in both in vitro and in vivo experimental models.
